# Recent advances in the diagnosis and treatment of bladder cancer

**DOI:** 10.1186/1741-7015-11-13

**Published:** 2013-01-17

**Authors:** Grace Cheung, Arun Sahai, Michele Billia, Prokar Dasgupta, Muhammad S Khan

**Affiliations:** 1 Urology Centre, Guy's Hospital, Guy's and St Thomas' NHS Trust, London, SE1 9RT, UK; 2 Medical Research Council (MRC) Centre for Transplantation, King's College London, King's Health Partners, Guy's Hospital, London, SE1 9RT, UK

**Keywords:** bladder cancer, cystoscopy, narrow-band imaging, photodynamic diagnosis, radical cystectomy, urinary markers

## Abstract

Bladder cancer is the commonest malignancy of the urinary tract. In this review, we look at the latest developments in the diagnosis and management of this condition. Cystoscopy and urine cytology are the most important tools in the diagnosis and follow-up of bladder cancer. Various alternatives have been investigated, either to reduce the frequency of cystoscopy, or improve its sensitivity for detection of tumors. These include urine-based markers and point-of-care tests. Narrow-band imaging and photodynamic diagnosis/blue-light cystoscopy have shown promise in improving detection and reducing recurrence of bladder tumors, by improving the completion of bladder resection when compared with standard resection in white light. The majority of patients with a new diagnosis of bladder cancer have non-muscle-invasive bladder cancer, which requires adjuvant intravesical chemotherapy and/or immunotherapy. Recent developments in post-resection intravesical regimens are discussed. For patients with muscle-invasive bladder cancer, both laparoscopic radical cystectomy and robot-assisted radical cystectomy have been shown to reduce peri-operative morbidity, while being oncologically equivalent to open radical cystectomy in the medium term. Bladder-preserving strategies entail resection and chemoradiation, and in selected patients give equivalent results to surgery. The development, advantages, and disadvantages of these newer approaches are also discussed.

## Introduction

Bladder cancer is the commonest malignancy of the urinary tract, with the incidence being four times higher in men than in women [1]. Much has changed in the diagnosis and management of bladder cancer over the past 5 to 10 years. Interestingly, in Europe, mortality rates appeared to have declined in the past decade, by approximately 16% in men and 12% in women [2]. Although cystoscopy remains a fundamental investigative tool in the detection and surveillance of bladder cancer, *s*mall papillary tumors or carcinoma *in situ *(CIS) can be easily missed by standard white-light cystoscopy (WLC), which may account for early recurrence. This has led to the development of newer technologies such as narrow-band imaging (NBI) cystoscopy and photodynamic diagnosis (PDD) [3]. Several molecular urinary tests to help in detection of bladder cancer have been marketed over the years. Although initially promising, none has been sufficiently sensitive or specific to prevent cystoscopic surveillance [4]. These new aspects of diagnosis will be discussed in separate sections below.

Approximately 75 to 85% of patients will have disease confined to the mucosa (Ta) or submucosa (T1), that is, non-muscle invasive bladder cancer (NMIBC), which was previously known as 'superficial' bladder cancer. NMIBC requires adjuvant intravesical chemotherapy and/or immunotherapy. The type and number of intravesical instillations given depend on numerous factors, including grade, stage, multifocality of the tumor, and tolerability. NMIBC has a high risk of recurrence and a variable risk of progression. CIS (tumor *in situ*; Tis) is a high-risk disease for muscle-invasion. CIS and T1 disease that is refractory to bacille Calmette-Guérin (BCG) immunotherapy, high-grade recurrent T1 disease, muscle-invasive bladder cancer (MIBC) (stage >T2), or high-volume disease that cannot be managed endoscopically should be managed with radical cystectomy [5]. Minimally invasive cystectomy techniques, such as laparoscopy (with or without robot assistance) have been developed [6,7]. Bladder-preservation strategies to treat MIBC also seem to be effective treatments, and with proper patient selection can be equivalent to radical surgery [8]. This review will cover recent advances in the diagnosis and management of non-metastatic bladder cancer.

## Classification

Classification of bladder cancer is important to determine the appropriate treatment strategy and predict outcomes. The WHO grading of bladder cancer has changed, with the 2004 grading system incorporating a range of histologic descriptions such as urothelial papilloma (completely benign lesion), papillary urothelial neoplasm of low malignant potential (PUNLMP), and low-grade and high-grade cancer, rather than the previous three grades of well (G1), moderately (G2) or poorly differentiated (G3) papillary urothelial carcinoma [9,10]. The TNM (tumor, node, metastasis) classification has generally remained the same, with some minor changes to the nodal classification made in the last report in 2009 [11].

### Diagnosis

#### Cystoscopy

Standard WLC has been used to detect and resect bladder tumors for several decades. New technologies have been developed to improve the quality of cystoscopy and transurethral resection of bladder tumor (TURBT) that is currently achieved, with the aim of preventing disease recurrence and progression.

#### Photodynamic diagnosis/blue-light cystoscopy

**Figure 1:**
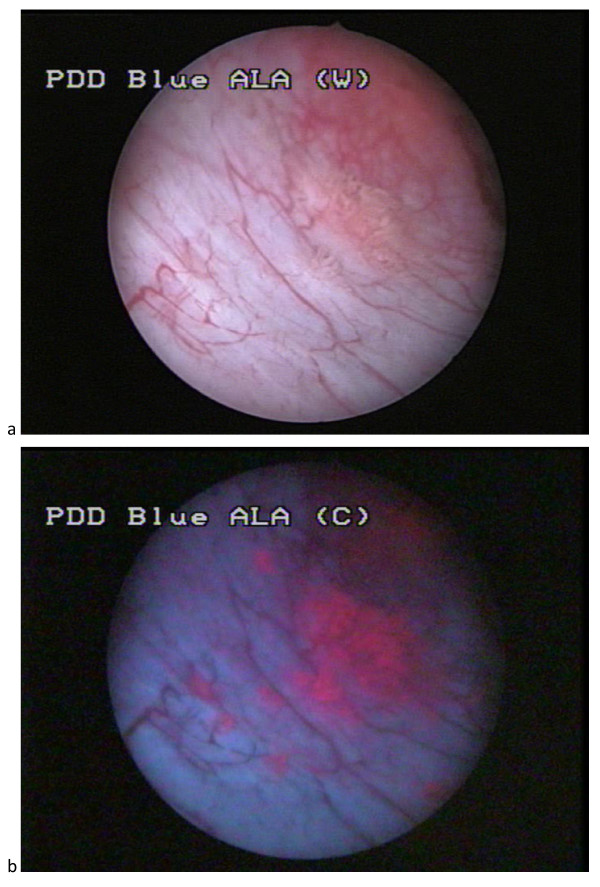
**(a) White-light and (b) blue-light endoscopic image of flat lesions adjacent to a small papillary tumor**. The photographs were produced specifically for this manuscript.

PDD improves the detection rates for inconspicuous bladder cancer. 5-aminolevulinic acid (5-ALA) dye, or its hexyl ester hexaminolevulinate (HAL; Hexvix^®^; Photocure ASA, Oslo, Norway), is instilled into the bladder and absorbed by dysplastic tissue, enabling photosensitization. The abnormal tissue emits a red color under blue reference light. Normal tissue appears blue (Figure 1). PDD is recommended to aid diagnosis during initial TURBT, and in patients with positive urine cytology but negative WLC results, for the assessment of tumor recurrences in patients not previously assessed with PDD. It is also recommended in the initial follow-up of patients with CIS or multifocal tumors [12]. A meta-analysis and systematic review has shown that blue-light cystoscopy (BLC) detects more bladder tumors than WLC, including more high-risk tumors. Based on four randomized controlled trials (RCTs) reporting clinical effectiveness, BLC with 5-ALA used at the time of TURBT facilitates a more complete resection and prolongs recurrence-free survival. However, the administration of adjuvant intravesical therapy varied across the four RCTs analyzed, which may have further diminished the benefit of PDD [13]. As yet, PDD has not been shown to prevent progression or improve survival.

#### Narrow-band imaging

NBI cystoscopy enhances the fine structure of the bladder mucosal surface without the use of dyes. The longer wavelengths of light enable deeper penetration. Whereas BLC requires pre-operative instillation of photosensitizing agents via a urethral catheter, NBI cystoscopy does not require extra invasive steps. It can also be performed using a flexible cystoscope, and therefore is convenient in an outpatient setting. NBI cystoscopy improves detection of recurrent NMIBC over standard WLC, with a comparative false-positive rate [14]. TURBT performed with NBI reduces the recurrence risk of NMIBC by at least 10% at 1 year [15]. As yet, there have been no clinical trials comparing NBI cystoscopy versus WLC or BLC.

#### Urinary markers

The most widely adopted non-invasive urine test is cytology, which has good specificity and sensitivity for the detection of high-grade tumors, but poor sensitivity for low-grade tumors [16], and has a delay in result availability. Numerous urinary markers have been investigated (Table 1), with the aim of reducing frequency of cystoscopy [4]. Several are commercially available, but none has been adopted into routine standard of care, owing to poor sensitivity and/or expense. These markers may serve as an adjunctive diagnostic test in cases where urine cytology is equivocal.

**Table 1:** Summary of main urinary markers

Markers	Overall sensitivity, %	Overall specificity, %	Sensitivity for high-grade tumors, %	Point-of-care test	Interference^b^	Comments
Aneuploidy detection kit^c^	30 to 72	63 to 95	66 to 70	No	No	Expensive and laborious

Microsatellite analysis	58	73	90	No	No	Expensive and laborious

Gene microarray	80 to 90	62 to 65	80	No	No	Expensive and laborious

Monoclonal antibodies^d^	76 to 85	63 to 75	67 to 92	No	Yes	Good sensitivity in low-grade tumors, affected by BCG

NMP 22	49 to 68	85.8 to 87.5	75 to 83	Yes	Yes	Low sensitivity, affected by benign conditions

Physician-use BTA immunoassay**^e^**	57 to 83	68 to 85.7	61.5	Yes	Yes	Low sensitivity, affected by benign conditions and BCG

Quantitative laboratory BTA immunoassay**^f^**	53 to 91	28 to 83	77	No	Yes	Low sensitivity, affected by benign conditions and BCG

Cytokeratins	12 to 85	75 to 97	33 to 82	No	Yes	Low sensitivity, affected by benign conditions and BCG

Survivin	53 to 90	88 to 100	50	No	No	Low sensitivity, expensive and laborious

Abbreviations: BCG, bacille Calmette-Guérin; BTA, bladder tumor-associated antigen; NMP, nuclear matrix protein

^a^Adapted from Expert Review of Anticancer Therapy, June 2010, Vol. 10, No. 6, Pages 787-790 with permission of Expert Reviews Ltd.

**^b^**By BCG instillations and other bladder conditions.

^c^UroVysion®; Abbott Molecular Inc , Des Plaines, IL, USA.

^d^Immunocyt/uCyt+™; DiagnoCure Inc., Québec, Canada.

^e^BTA *stat*^®^; Polymedco Inc., Cortlandt Manor, NY, USA.

^f^BTA TRAK™; Polymedco Inc.

Fluorescence *in situ *hybridization (FISH) can be used to detect urinary cells that have chromosomal abnormalities consistent with a diagnosis of bladder cancer. For example, one commercial kit (UroVysion^®^; Abbott Molecular Inc , Des Plaines, IL, USA) uses fluorescently labeled DNA probes to detect aneuploidy in chromosomes 3, 7, and 17, and loss of the 9p21 locus of the P16 tumor suppressor gene . [17]. In most comparative studies, FISH outperformed cytology across all stages and grades of bladder cancer [18-20]. However, there is a high false-positive rate, and the test is expensive, therefore, FISH results may be used mainly when urine cytology results are inconclusive. Furthermore, in a recent study, FISH was shown to identify patients at risk for tumor recurrence and progression during BCG immunotherapy [21]; this information could be used to counsel patients about alternative treatment strategies.

Nuclear mitotic apparatus protein (NMP)22 is another marker that can be detected in voided urine. A point-of-care tumor-marker test (NMP22® Bladderchek®; Stellar Pharmaceuticals, London, ON, USA) has been approved by the USA Food and Drugs Administration for bladder-cancer surveillance. It was shown that for patients with negative results for both cystoscopy and NMP22 Bladderchek test, very few cancers were missed, and cytology did not yield any further useful diagnostic information [22]. In cases where physicians would perform a cystoscopy even if there was a low risk of progression (1%) or recurrence (5%), the use of NMP22 did not aid clinical decision-making [23]; however, if the thresholds were increased to 3% and 8% for progression and recurrence, respectively, NMP22 could help distinguish which patients would need cystoscopy or not.

### Treatment

#### Non-muscle invasive bladder cancer

TURBT is the first-line treatment for patients with NMIBC. Unfortunately, the high rate of recurrence and progression after TURBT necessitates the use of adjuvant treatments [5,24,25]. This entails instillation of chemotherapeutic, usually mitomycin-C (MMC), or immunotherapapeutic agents such as BCG, either alone or in various combinations. A single dose of intravesical chemotherapy given after TURBT but on the same day significantly reduced the odds of tumor recurrence by 39% in patients with tumors with a low risk of recurrence and progression [26], although the technique is significantly underutilized [27]. Intravesical BCG is the standard treatment for high-grade NMIBC and CIS, and should be given as a maintenance schedule [28]. Unfortunately, some patients are unable to tolerate the side-effects of hematuria or of irritative lower urinary-tract symptoms, and/or are refractory to treatment.

#### Bacille Calmette-Guérin treatment and electromotive drug administration of mitomycin C

Di Stasi *et al*. reported that intravesical, sequential BCG, followed by electromotive administration (EMDA) of MMC (EMDA-MMC) given to patients with high-risk superficial bladder led to higher disease-free interval, lower recurrence and progression rates, and improved overall survival (OS) and disease-specific survival (DFS) rates, compared with BCG alone. The treatment schedule consisted of a 9-week induction course, comprising two weekly instillations of BCG and one of EMDA-MMC, repeated three times. If patients were disease-free after 3 months, they were placed on a monthly maintenance schedule, receiving EMDA-MMC for 2 months, followed by BCG for 1 month, with this cycle again repeated three times [29]. The authors proposed that BCG-induced inflammation might increase the permeability of the bladder mucosa, allowing MMC to reach the target tissue more easily and exert its anticancer effect. More recently, the same group showed that intravesical EMDA-MMC before TURBT is feasible and safe. Importantly, it reduced recurrence rates and enhanced the disease-free interval compared with TURBT alone or with intravesical passive diffusion of MMC after TURBT [30]. However, as yet, no other institutions have been able to reproduce these results. Our institution introduced sequential BCG/EMDA-MMC as our standard regimen in high-risk NMIBC in 2009. Of 62 patients who were treated, 10 did not respond to treatment. Of the remaining 52 patients, 21 patients have completed the 12-month follow-up, and 17 of these remain disease-free [31].

#### Hyperthermic mitomycin C

A combined regimen of intravesical MMC and microwave-induced bladder wall hyperthermia (HT) for Ta/T1 bladder cancer was first introduced in 1995, and endoscopic and histological analysis has shown that this combination can induce necrosis of transitional cell tumors [32]. A small RCT comparing MMC-HT versus MMC alone has since shown a significantly improved DFS rate at 10 years [33]. A systematic review suggested a 59% relative reduction in NMIBC recurrence when MMC-HT was administered, compared with MMC alone; however, this review was limited by the small number of RCTs available for inclusion. The authors speculated that in the future, MMC-HT might become the standard therapy for high-risk patients with recurrent tumors, patients who are unsuitable for radical cystectomy, and patients for whom BCG treatment is contraindicated [34].

#### Intravesical gemcitabine

Gemcitabine has recently been introduced as a chemotherapeutic agent for metastatic bladder cancer [35]. Administration of intravesical gemcitabine for NMIBC has been explored and deemed to be safe, with an acceptable side-effect profile. A Cochrane review of the current evidence base of randomized trials was limited because of the heterogenous patient populations. Patients with high-risk NMIBC were found to have increased risk of recurrence and progression when receiving intravesical gemcitabine compared with BCG; however, high-risk BCG-refractory patients treated with intravesical gemcitabine experienced fewer recurrences than those receiving BCG. Multiple doses would be required, rather than a single shot after surgery [36]. The South-West Oncology Group are currently trialing gemcitabine (6-weekly instillation s followed by monthly maintenance for 12 months) in patients refractory to BCG. At 1 year, only 28% of evaluable patients had a durable response [37].

#### Muscle-invasive bladder cancer

##### Minimally invasive techniques in radical cystectomy

Open radical cystectomy (ORC) is the current gold-standard treatment for MIBC and for high-risk recurrent NMIBC. Ideally, all patients with MIBC should receive platinum-based neo-adjuvant chemotherapy [38,39]. ORC has a peri-operative complication rate of 25 to 62% [40]. Therefore, minimally invasive techniques in radical cystectomy have been explored.

The majority of the existing data comprises cohort studies. The advantages of laparoscopic radical cystectomy (LRC) include decreased blood loss, reduced postoperative pain, early return of bowel function, and shorter hospital stay [6,41]. However, the data should be interpreted with caution, given the problem of selection bias in most series. Overall, current evidence suggests that LRC has good early oncologic outcomes with low morbidity in large cohorts with up to 5 years follow-up [42]. Nevertheless, LRC is considered an advanced laparoscopic procedure, because it has multiple difficult steps and fewer degrees of freedom of movement compared with ORC.

In 2001, the da Vinci^® ^robot (Intuitive Surgical Inc., CA, USA) was introduced as an innovative system for minimally invasive surgery. The view of the operative field is improved by binocular three-dimensional high-definition endoscopic vision. 'Endowrists' on the tip of each instrument can reproduce the movements of the human hand. A small RCT by Nix *et al*. of robot-assisted radical cystectomy (RARC) versus ORC showed that RARC produced a reduction in operative blood loss, time for return of bowel function, and analgesic requirements, compared with ORC, with equivalent lymph-node (LN) yields [7]. A prospective cohort study showed fewer major complication rates at 30 and 90 days post- RARC compared with ORC [43]. The short-term outcomes of RARC are promising, with an OS rate of 70 to 90% during 2 to 3 years of follow-up [44-47]. The International Robotic Cystectomy Consortium comprises 18 institutions, which have reported comparable rates to ORC for LN yields and positive surgical margin rates [48,49]. To date, 1,200 cystectomies have been recorded on their collaborative database [50].

Most urologists performing LRC or RARC advocate performing the cystectomy and LN dissection intracorporeally, with subsequent extracorporeal urinary diversion via a lower midline incision. Increasing experience has enabled intra-corporeal reconstruction of urinary diversion, whether this be by ileal conduit or orthotopic neobladder formation. Clearly, the learning curve is steep. Operative times are longer, although patients have lower inpatient narcotic requirements and comparable short-term clinical outcomes to extra-corporeal diversion [51-53].

However, there is a distinct lack of RCTs comparing RARC with ORC. Several that are currently under way, including the randomized CORAL (Randomised Control Trial of Open, Robotic and Laparoscopic Radical Cystectomy) trial [54], a trial in the University of Texas Health Science Centre, USA (Open Versus Robotic-Assisted Radical Cystectomy: A Randomised Trial) [55], and the BOLERO (Bladder Cancer: Open Versus Laparoscopic or Robotic Cystectomy) trial at Cardiff University, UK [56]. The long-term outcomes of the first cohort of patients who underwent RARC should be available in the next 1 to 2 years.

##### Bladder preservation

Strategies for bladder preservation have also been investigated. A phase III trial of chemotherapy (fluorouracil plus mitomycin) combined with radiotherapy was shown to improve the 2-year DFS rate compared with radiotherapy alone, and it also decreased the salvage cystectomy rate, with good long-term bladder function [8]. Long-term data from Massachusetts General Hospital, USA, has shown that combined multi-modal therapy in the form of concurrent cisplatin-based chemotherapy and radiotherapy after maximal TURBT achieves complete response and preserves the native bladder in more than 70% of patients, while offering long-term survival rates comparable with contemporary cystectomy series [57]. However, a number of different protocols were used in this centre, and so the optimal therapy regimen is still uncertain. However, these studies suggest that this approach could be a real alternative to radical surgery in select patients with muscle-invasive disease.

## Conclusions

Several new techniques and developments have been introduced in recent years to improve the diagnosis and management of bladder cancer. Technological advances have enabled enhanced cystoscopy, with BLC increasing detection of bladder tumors and improving quality of tumor resection. Although many types of urinary markers have been explored, none as yet exists with sufficient specificity or sensitivity to replace regular cystoscopic surveillance of NMIBC. Various intravesical therapies for NMIBC have been investigated, including MMC-HT, BCG in combination with EMDA-MMC and intravesical gemcitabine. Minimally invasive radical cystectomy has been increasing in popularity over the past 10 years. The eagerly anticipated results of collaborative international efforts and randomized trials will improve the quality of existing evidence for these newer surgical approaches. While the long-term benefits of RARC are awaited, ORC remains the gold-standard treatment for MIBC. For patients who wish for bladder preservation, TURBT with combination chemotherapy and radiotherapy regimens has shown equivalent outcomes to radical surgery for selected patients.

## Abbreviations

BCG: bacille Calmette-Guérin; LRC: laparoscopic radical cystectomy; MIBC: muscle-invasive bladder cancer; MMC: mitomycin C; MMC-HT: mitomycin C with hyperthermia; NBI: narrow-band imaging; NMIBC: non-muscle-invasive bladder cancer; ORC: open radical cystectomy; RARC: robot-assisted radical cystectomy; RCT: randomized controlled trial.

## Competing interests

Arun Sahai and Prokar Dasgupta have received financial support from the Department of Health via the National Institute for Health Research (NIHR) comprehensive Biomedical Research Centre award to Guy's & St Thomas' NHS Foundation Trust in partnership with King's College London and King's College Hospital NHS Foundation Trust.

## Authors' contributions

AS and MSK conceived the outline of the manuscript, and GC and MB wrote the initial version of the manuscript. AS, PD, and MSK critically reviewed the manuscript. All authors read and approved the final manuscript.

## Authors' information

GC is a research fellow in Urology at Guy's Hospital. AS is an academic clinical lecturer and specialist trainee in Urology at Guy's Hospital. MB is a robotic and minimally invasive fellow in Urology at Guy's Hospital. PD is Professor of Urological Innovation for the Medical Research Council (MRC) Centre for Transplantation, King's College London, and a Consultant Urological Surgeon at Guy's Hospital. MSK is a Reader in Urology at King's College London and Consultant Urological Surgeon at Guy's Hospital.

## Pre-publication history

The pre-publication history for this paper can be accessed here:

http://www.biomedcentral.com/1741-7015/11/13/prepub
